# Transcriptional-Readthrough RNAs Reflect the Phenomenon of “A Gene Contains Gene(s)” or “Gene(s) within a Gene” in the Human Genome, and Thus Are Not Chimeric RNAs

**DOI:** 10.3390/genes9010040

**Published:** 2018-01-16

**Authors:** Yan He, Chengfu Yuan, Lichan Chen, Mingjuan Lei, Lucas Zellmer, Hai Huang, Dezhong Joshua Liao

**Affiliations:** 1 Key Lab of Endemic and Ethnic Diseases of the Ministry of Education of China in Guizhou Medical University, Guiyang 550004, Guizhou, China; 2 Department of Biochemistry, China Three Gorges University, Yichang City 443002, Hubei, China; 3 Hormel Institute, University of Minnesota, Austin, MN 55912, USA; lchen@hi.umn.edu (L.C.); mlei@hi.umn.edu (M.L.); 4 Masonic Cancer Center, University of Minnesota, 435 E. River Road, Minneapolis, MN 55455, USA; lzellmer@umn.edu; 5 School of Clinical Laboratory Science, Guizhou Medical University, Guiyang 550004, Guizhou, China; 6 Department of Pathology, Guizhou Medical University Hospital, Guiyang 550004, Guizhou, China

**Keywords:** chimeric RNA, fusion gene, transcriptional readthrough, *cis*-splicing, *trans*-splicing, reverse transcription, polymerase chain reactions

## Abstract

Tens of thousands of chimeric RNAs, i.e., RNAs with sequences of two genes, have been identified in human cells. Most of them are formed by two neighboring genes on the same chromosome and are considered to be derived via transcriptional readthrough, but a true readthrough event still awaits more evidence and *trans*-splicing that joins two transcripts together remains as a possible mechanism. We regard those genomic loci that are transcriptionally read through as unannotated genes, because their transcriptional and posttranscriptional regulations are the same as those of already-annotated genes, including fusion genes formed due to genetic alterations. Therefore, readthrough RNAs and fusion-gene-derived RNAs are not chimeras. Only those two-gene RNAs formed at the RNA level, likely via *trans*-splicing, without corresponding genes as genomic parents, should be regarded as authentic chimeric RNAs. However, since in human cells, procedural and mechanistic details of *trans*-splicing have never been disclosed, we doubt the existence of *trans*-splicing. Therefore, there are probably no authentic chimeras in humans, after readthrough and fusion-gene derived RNAs are all put back into the group of ordinary RNAs. Therefore, it should be further determined whether in human cells all two-neighboring-gene RNAs are derived from transcriptional readthrough and whether *trans*-splicing truly exists.

## 1. Introduction

In 2007, the ENCODE (The Encyclopedia of DNA Elements) pilot project reported its identification and analysis of functional elements in 1% of the human genome [1,2]. In this report, it was estimated that RNAs from 65% of human genes are fused to another gene’s RNA to form a new RNA that contains sequences of two genes and is called “chimeric RNA” or chimera. Interestingly, most of these chimeras are formed by RNAs from two neighboring genes on the same chromosome [1,2]. Since this ENCODE report, high-throughput RNA sequencing technology has swiftly spread over all biomedical research and has led to the identification of tens of thousands of chimeric RNAs and other forms of noncolinear RNAs [3,4], as summarized by us previously [5,6]. This number is astonishing, considering that the human genome contains only about 20,000 protein-coding genes [7,8,9,10,11,12,13,14], although the number of genes may be much larger if noncoding genes are included and if readthrough genomic loci are considered as newly-identified genes and are included, as we have suggested before [6]. Many fusion RNAs derived from fusion genes formed due to genetic alterations [15,16,17,18], seen mainly in genetic diseases and tumors [19,20,21,22,23,24], have also been identified and are, peculiarly, renamed as chimeras, as they also contain sequences of two genes [5,6]. This reclassification of fusion RNAs to tout their novelty and importance seems unnecessary, as they belong to an ancient research sphere the importance of which has already been recognized for roughly six decades, since 1959, when the Philadelphia chromosome and its-encoded fusion genes were identified [25,26,27,28,29,30].

Despite the sheer number of chimeras identified, unfortunately neither the ENCODE’s report nor most other relevant studies have disclosed the procedural and mechanistic details on how most chimeras might be formed. Since it is well known that transcription of genes occasionally does not stop at the canonical termination site but instead reads into the downstream gene [31,32,33], it is speculated that transcriptional readthrough may be a major mechanism for those two-neighboring-gene RNAs, albeit other mechanisms remain possible, such as *trans*-splicing that splices two RNAs into one. While the “readthrough” assumption is very reasonable and has been widely accepted, there is little irrefutable experimental proof to validate that a readthrough has indeed happened during formation of most two-neighboring-gene RNAs, and only a few such RNAs have received tenable evidence, because detection of a not-yet-spliced precursor transcript is difficult [34,35]. This in turn is because transcription is a transient procedure and splicing of the resulting transcript to a mature RNA ensues nearly at the same time as the start of transcription and is terminated almost as transcription is finished, making it difficult for researchers to determine what events transpired during this short spell [35]. Some relevant questions, such as why the transcription does not end as it should at the upstream gene, still remain inscrutable as well for most such RNAs. Moreover, although “chimeric RNA” means that an RNA consists of sequences of two different genes, the reality is that it has never been lucidly defined, and a variety of noncolinear RNAs have all been called “chimeric RNA” [6]. This is, in turn, because “what is a gene” remains an unanswered question, and many researchers, including us, consider that “gene” should be redefined at the RNA level in the post ENCODE era [5,6,36,37,38,39,40], and thus consider two-RNA RNAs as chimeras as well. All these problems have made many researchers befuddled and have gutted not only research on authentic chimeric RNAs per se but also research into ordinary colinear RNAs. 

In this perspective article, we elaborate on our contemplation and reflection on the designation and classification of noncolinear RNAs, including those RNAs that contain sequences of two genes or contain transcripts from both DNA strands of a gene, for our contemporaries in the RNA research bailiwick to consider and debate. Only messenger RNAs (mRNAs) and long, i.e., larger than 200 nucleotides [41,42,43], noncoding RNAs are of concern, while those short RNAs that are generally esteemed to function as regulatory elements of genes are left out, in part because we are not aware of any chimeric short regulatory RNAs, which are typically 20 nucleotides in length. We refer to the DNA strand that harbors the gene as the Watson strand and its opposite strand of the DNA double helix as the Crick strand, to avoid confusion, since in the literature different researchers define Watson and Crick strands differently, whereas the RNAs transcribed from the Watson and Crick strands of a gene are referred to as sense and antisense, respectively.

## 2. There Are Different Types of Long Noncolinear RNAs

While probably less than 5% of the human genes (including all mitochondrial genes) contain only a single exon, and, thus, their transcripts do not need to undergo *cis*-splicing to produce mature RNA, transcripts from over 95% of human genes need to be *cis*-spliced to remove intron(s) and to join exons together for the formation of mature RNAs [34,35,38,44]. Because of the removal of intron sequence(s), mature RNAs are no longer as continuous as the parental genes’ sequences, but they still have the same 5’-to-3’ orientation and, thus, are colinear, which in our opinion includes circular RNAs a well. However, there remains a large number of noncolinear RNAs, but mainly in evolutionarily-lower organisms, such as bacteria and other prokaryotic or unicellular eukaryotic organisms [45,46,47,48,49,50]. Nevertheless, noncolinear mature RNAs have also been reported in the cells of human, mouse, and rat origins, which to our knowledge include the following types:

1. RNAs with sequences of two different genes, which occur in two separate ways, i.e., (1) the two genes are adjacent to each other on the same chromosome; and (2) the two genes are located on two different chromosomes. Theoretically, there should also be many RNAs in which the two genes are on the same chromosome but are far away from each other, too far away for a transcriptional-readthrough to occur, but, unfathomably, there are few, if any, such RNAs reported in the literature, to our knowledge.
2. RNAs that contain repeats of one or more exons [51,52,53], such as some RNA variants of human estrogen receptor α (*ERα*) [54,55,56], rat *Cot* [57,58,59], rat *Sns* [60], and rat *Sa* [61].
3. RNAs that contain both sense and antisense sequences of the same gene, with the drosophila mdg4 mRNA variant being best studied [62,63].

A caveat needs to be given that many genetic alterations, as often seen in genetic diseases and tumors [19,20,21,22,23,24], can also lead to the formation of the abovementioned three categories of RNA in pathological situations. Indeed, some genetic alterations can cause fusion of two genes into one [15,16,17,18], and the fusion gene can be transcribed to two-gene RNAs in the same way as other genes [5,6]. Similarly, some genetic alterations can also result in RNAs with duplicated exons or with antisense sequences. However, the RNAs caused by these genetic alterations are still colinear and, thus, are excluded, because they have a corresponding gene as a genomic parent and are produced in the same way as all colinear RNAs from all genes.

## 3. *Trans*-Splicing Remains as a Possible Mechanism for Formation of Chimeric and Other Noncolinear RNAs

Besides *cis*-splicing that is a biochemical reaction using one single RNA molecule as the substrate and producing one single mature RNA as the product, there is also *trans*-splicing, which is another biochemical reaction that uses two RNA molecules as the substrates but produces only one single mature RNA as the product [5,6,64,65]. Although *trans*-splicing is a common event in some unicellular organisms, in some mitochondria of evolutionarily-lower eukaryotes, and in chloroplasts of some plants [45,46,47,48,49,50], it is also considered by many researchers to occur as a mechanism for the formation of some chimeric RNAs and other forms of noncolinear RNAs in evolutionarily-higher animals [15,66,67,68,69,70,71,72,73,74]. For example, a human *KLK4* RNA [75] was found to contain both sense and antisense sequences, and some RNA variants of *ERα* [54,55,56] and *Sp1* [76,77] were reported to bear duplicated exons. In normal human endometrium and in some human uterine tumors, a chimeric RNA involving a *JAZF1* sequence from 7p15 and a *JJAZ1* sequence from 17q11 has been reported to be derived via a *trans*-splicing like mechanism [78,79,80], although it has been known that these uterine tumors bear a *JAZF1*-*JJAZ1* fusion gene at high frequencies [78,81,82,83,84,85]. There are other reported chimeric RNAs in human cells that are not associated with a fusion gene, such as the *CCND1*-*Trop2* [25,86], *FAS*-*ERα* [87], *CYP3A43*-*CYP3A4* [88], *CYP3A43*-*CYP3A5* [88] and *Yq12*-*CDC2L2* [89] RNAs as well as an *ACTAT1* RNA that contains sequences from both chromosomes 1 and 7 [90,91,92,93]. A more complicated case is the seven mouse *Msh4* RNA variants, which together involve sequences from a total of four different chromosomes, and some of which involve both sense and antisense sequences of one of the genomic loci [94]. However, although these noncolinear RNAs were considered to be derived from a *trans*-splicing or a *trans*-splicing-like mechanism, unimpeachable evidence for a *trans*-splicing event in the formation of these RNAs, and the procedural and mechanistic details of the splicing, are still lacking. After a decade since the initial reports on most of these RNAs, such as the *JAZF1*-*JJAZ1* RNA, we do not possess information about the procedural and mechanistic details of their *trans*-splicing to corroborate that they are really formed at the RNA level and are not technical artifacts or are not transcribed from a fusion gene. On the other hand, more publications continue emerging to report [95] or summarize [3,69,70] such *trans*-splicing related chimeras or other noncolinear RNAs. Moreover, many bioinformatic experts are establishing different algorithms to cull chimeras from different sets of high-throughput sequencing data [96,97,98,99,100,101,102,103,104], although all these data sets contain many spurious sequences, as we and others have pointed out [5,6,64,105,106,107,108,109,110,111,112,113,114,115,116,117,118,119,120,121,122,123,124,125,126,127,128,129,130,131,132,133,134,135]. This situation is worrisome to us. 

Although it should be a requirement to show more-concrete evidence for the true existence of *trans*-splicing in evolutionarily-higher animal species, such as in the human, rat, and mouse, there are technical constraints hindering such studies [6,105]. For example, splicing is initiated and finished too quickly to study its detail, as aforementioned. In addition, the reported detection of the abovementioned RNAs all involved reverse transcription (RT) and polymerase chain reactions (PCR), which are techniques that easily create spurious results, as we and others have repeatedly described before, due to template-switching, mis-priming, self-priming, DNA or complementary DNA (cDNA) damage, and PCR-reconditioning, among other reasons [5,6,64,105,106,107,108,109,110,111,112,113,114,115,116,117,118,119,120,121,122,123,124,125,126,127,128,129,130,131,132,133,134,135]. Therefore, approaches without involvement of RT and PCR are needed to minimize technical artifacts for indisputable evidence and to obtain procedural and mechanistic details of the presumed *trans*-splicing. RNA protection assay [136,137,138], or the cDNA protection assay established by us [105], is currently the best approach for this purpose, to our knowledge.

## 4. Some Human Genomic Loci Are Crowded Gene Habitats

**Figure 1:**
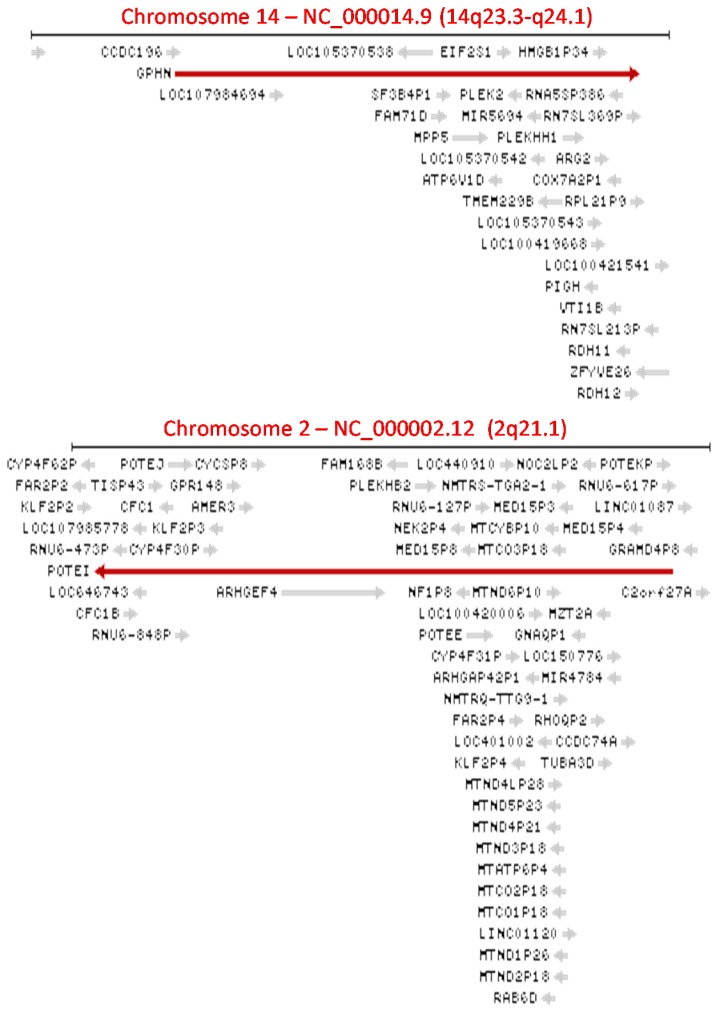
Images copied from the National Center for Bioinformation of the United States (NCBI) database illustrating that some human genomic loci are crowded gene habitats. Within the *GPHN* gene (the long red arrow in the top image) on the plus DNA strand (arrow to the right) of the 14q23.3–24.1 region, there are also many other genes encoded not only by the same plus strand (short grey arrows to the right) but also by the minus strand (short grey arrows to the left). Similarly, within the *POTEI* gene (the long red arrow in the bottom image) on the minus DNA strand (arrow to the left) of the 2q21.1 region, there are also many other genes encoded not only by the same minus strand (short grey arrows to the left) but also by the plus strand (short grey arrows to the right). Some of these genes are temporarily annotated with “LOC” (locus) and a number, since they have not yet been characterized.

In the human genome, genes are not evenly dispersed over chromosomal DNA. Some genomic loci are very crowded gene habitats, such as the 14q23.3-24.1 and 2q21.1 chromosomal regions (Figure 1), while other genomic regions harbor very few genes. In those crowded loci, “a gene contains gene(s)” or “gene(s) within a gene” is a common phenomenon [38]. For example, both the Watson and Crick strands of the *GPNH* gene or the *POTEI* gene encode many other genes, making the *GPNH* or *POTEI* a readthrough gene whose precursor transcript contains many other genes; thus, both are examples of “a gene contains gene(s)” or “gene(s) within a gene” (Figure 1). The genes within the *GPNH* or *POTEI* include not only protein-coding ones but also noncoding ones and pseudogenes, and some of them have until now not yet been characterized and, thus, are temporarily annotated with “LOC” (stands for Locus) and a number (Figure 1). Therefore, the precursor transcript of the *GPNH* or *POTEI* gene can be considered as a readthrough one that spans over many genes, meaning that readthrough can occur to multiple, and not just two, consecutive genes in a genomic locus, although the sequences of the inside genes may be lopped off during *cis*-splicing and, thus, may not occur in a *GPNH* or *POTEI* RNA variant.

**Figure 2:**
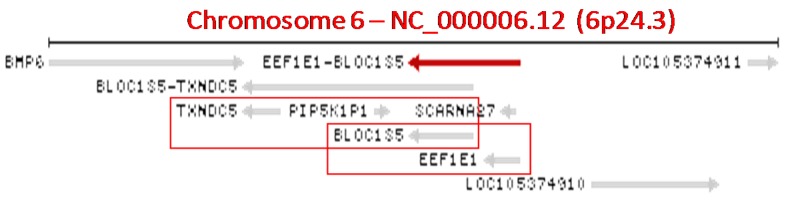
An image copied from the NCBI database illustrating that the *BLOC1S5* gene and its upstream gene *EEF1E1* on the minus strand of the 6p24.3 region together produce an *EEF1E1*-*BLOC1S5* RNA (red arrow to the left), while it and its downstream gene *TXNDC5* together produce a *BLOC1S5*-*TXNDC5* RNA (the long grey arrow to the left). Note that there is no *EEF1E1*-*BLOC1S5*-*TXNDC5* RNA shown in the image.

To our knowledge, the *CNTNAP2* (located at 7q35–36.1) and *PTPRD* (located at 9p24.1–9p23) genes, both being longer than 2.3 megabase-pairs, are among the largest genes in the human genome, while most other genes are smaller than one-tenth of this size. This means that a single transcription can read through at least 2.3 mega-nucleotides. Therefore, theoretically, transcription can also go through a genomic locus that contains several genes as long as it, for some reason, does not stop at a canonical transcription-termination site and as long as the transcription-distance is within 2.3 mega-nucleotides. Actually, there hitherto has been no evidence showing that a transcription cannot go beyond 2.3 mega-nucleotides. However, what is still inexplicable to us is that, to our knowledge, there has not been any mature RNA found known to possess sequences of three or more chromosomal genes, although we have found RNAs with sequences from three or four mitochondrial genes in some databases of expression sequence tags [65]. For instance, the NCBI (National Center for Bioinformation of the United States) database shows that on the minus strand of the human 6p24.3 region, the *BLOC1S5* gene and its downstream gene *TXNDC5* together produce a *BLOC1S5*-*TXNDC5* RNA, while the *BLOC1S5* and its upstream gene *EEF1E1* together produce a *EEF1E1*-*BLOC1S5* RNA (Figure 2 and Table 1). However, no RNA containing sequences of all three genes, i.e., no *EEF1E1*-*BLOC1S5*-*TXNDC5* RNA, has been reported so far. This conundrum, i.e., why there has not been a three-gene RNA reported, is bewitching and awaits exploration. 

**Figure 3:**
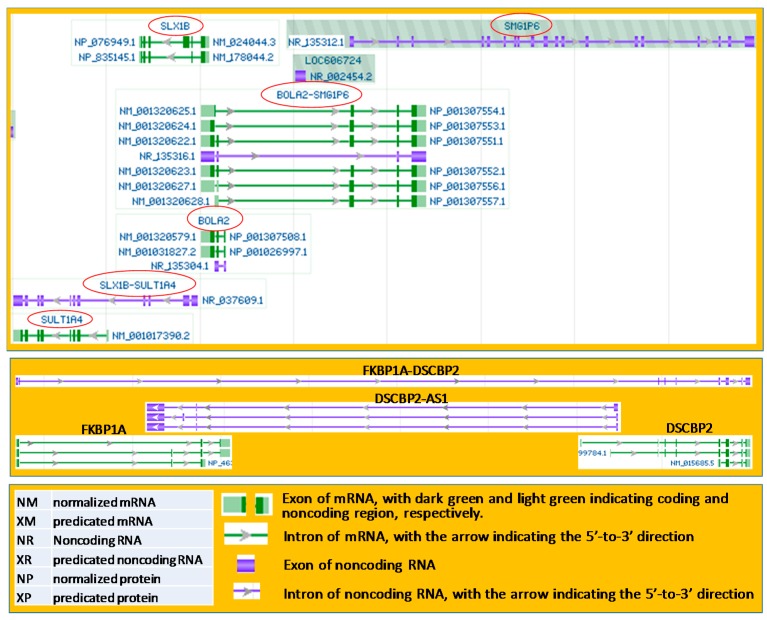
Illustrations copied and modified from the NCBI database showing transcripts from both strands of the DNA double helix. **Top panel**: the protein-coding *BOLA2* gene and the noncoding *SHG1P6* gene on the plus DNA strand together produce six *BOLA2*-*SMG1P6* messenger RNAs (mRNAs) and one noncoding *BOLA2*-*SMG1P6* RNA, while the protein-coding *SLX1B* and *SULT1A4* genes on the minus strand together produce a *SLX1B*-*SULT1A4* noncoding RNA. All genes or RNAs mentioned are highlighted with red circles. **Middle panel**: The protein coding *FKBP1A* and *SDCBP2* genes on the plus strand of the human 20p13 region together produce a *FKBP1A*-*SDCBP2* noncoding RNA, while the minus DNA strand of this region is also transcribed to three antisense (AS) RNAs that overlap, in a reverse-complementary manner, with an end of the *FKBP1A* and *SDCBP2* mRNAs. The overlaps can easily lead to creation of an artificial *FKBP1A*-*SDCBP2* cDNA during reverse transcription (RT) or PCR, as we described before [5,6,64,105]. **Bottom panel**: The NCBI database uses NM, XM, NR, XR, NP, and XP to indicate normalized mRNA, predicated mRNA, noncoding RNA, predicated noncoding RNA, normalized protein, and predicated protein, respectively, while it uses green and blue colors to indicate mRNA and noncoding RNA, respectively. The NCBI also uses boxes and lines to indicate exons and introns, respectively, with their lengths in proportion to the lengths of the exons or introns in the number of nucleotides (RNA) or base-pairs (DNA).

As another situation of the crowdedness of genomic loci, occasionally, both the plus and minus strands of the same genomic locus can produce RNAs that contain two genes’ sequences. For example, the plus strand of the human 16p11.2 region produces the *BOLA2*-*SMG1P6* RNA while the minus strand produces the *SLX1B*-*SULT1A4* RNA, as shown in the NCBI database (Figure 3, top panel). Moreover, when the opposite DNA strand does not encode gene(s), it may still produce antisense RNA(s) (Figure 3, middle panel).

## 5. Some Genes Are Encoded by the Same Genomic Locus with Their RNAs Sharing Exons

As aforementioned [5,6,36,37,38,39,40], “what is a gene” has become an unanswered question in the post ENCODE era. In our opinion, a long mature RNA should be regarded as a gene, regardless of whether it is protein-coding or noncoding and whether it is produced from a linear DNA or is produced solely at the RNA level without a corresponding genomic base [6,38]. Short noncoding RNAs should not be considered as genes because each of them, such as a microRNA, often is not unique and has many repeats in the 3.2–3.5 billion-base-pair sequence of the human genome [7,8,11]. 

**Figure 4:**
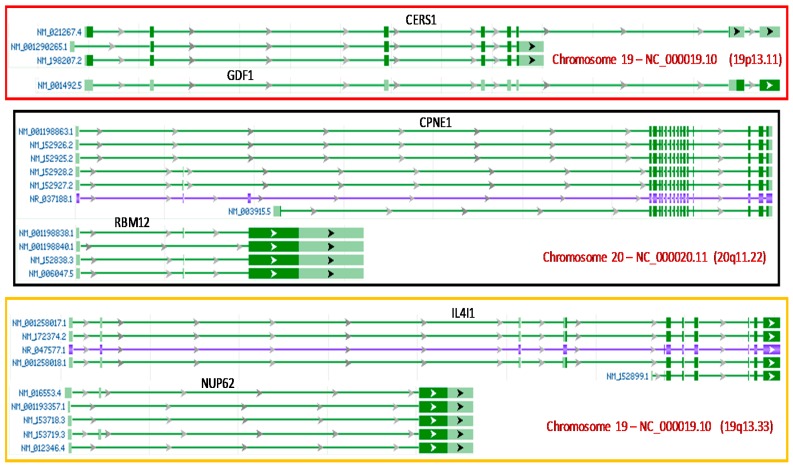
Images copied and modified from the NCBI database illustrating that one human genomic locus harbors two genes whose RNAs not only are transcribed from the same initiation site but also share exons. **Top panel**: The *CERS1* and *GDF1* genes are encoded by the same human genomic locus at the 19p13.11 region, and the *GDF1* mRNA is identical to the largest *CERS1* mRNA, but the same mRNA codes for different open reading frames (ORFs) for the *GDF1* and the *CERS1* genes. **Middle panel**: The *CPNE1* and *RBM12* genes are encoded by the same genomic locus at the human 20q11.22 region and are transcribed from the same initiation site. While the *CPNE1* transcripts may be *cis*-spliced to six mRNAs and one noncoding RNA, the *RBM12* transcripts may be *cis*-spliced to four mRNAs. The *CPNE1* RNAs share some exons with the *RBM12* RNAs. **Bottom panel**: The three mRNAs and one noncoding RNA of the *IL4I1* gene share some exons with the five mRNAs of the *NUP62* gene, and both genes locate at the same genomic locus in the human 19q13.33 region, with some RNAs of these two genes sharing the same transcription initiation site.

A protein, after it has been translated from an mRNA but before it is posttranslationally modified to different protein forms, should also be regarded as a gene, partly because in some special situations, one single mRNA sequence may be annotated as different genes in the NCBI, which is a special case of the “a gene contains gene(s)” situation or a special case of the crowdedness of some genomic loci. This situation can be reflected by the so-called “alternative reading frame (ARF)” of mRNAs, as seen in the mRNAs that are encoded by a single genomic locus called *INK4* and are translated to the p15, p16, and p19 tumor suppressor proteins in human and rodent cells [139,140]. As a better example, the *GDF1* mRNA (NM_001492.5) is identical to the longest mRNA (NM_021267.4) of the *CES1* gene, although it encodes different open reading frames (ORFs) when it is the *GDF1* mRNA than when it is one of the *CES1* mRNAs. This is because both *GDF1* and *CES1* genes reside at the same genomic locus (19p13.11) and are transcribed from the same initiation site, as illustrated in Figure 4. If we do not regard different proteins as different genes, the same mRNA-encoded *GDF1* and *CES1* can only be considered as the same gene. There are other similar cases in which two genes not only reside at the same genomic locus but are also transcribed from the same initiation site, with the RNAs of the two different genes sharing some exons. For example, the *RBM12* gene is within the *CPNE1* gene in the human 20q11.22 region, with the two genes sharing the same transcription initiation site and with two of the *RMB12*’s three exons also appearing in some *CPNE1* RNA variants (Figure 4). The relationships between the *IL4I1* and *NUP62* genes, and between their RNAs, are the same as those between the *CPNE1* and *RBM12* genes, and between their RNAs (Figure 4).

**Figure 5:**
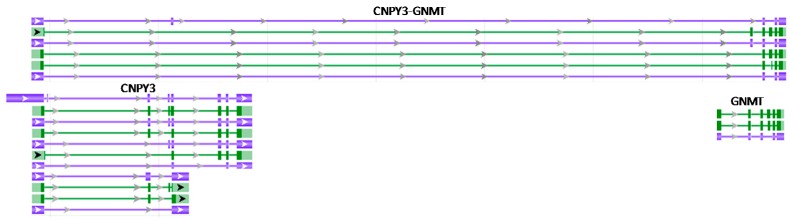
An illustration copied and modified from the NCBI database showing multiple mRNAs and noncoding RNAs of the *CNPY3*, *GNMT,* and *CNPY3-GNMT* genes in the human 6p21.1 region.

Many long mature RNAs that encompass sequences of two neighboring genes can be protein-coding or noncoding, regardless of whether their 5’ or 3’ partner gene encodes mRNA(s) or noncoding RNA(s). For instance, the *CNPY3* gene and its downstream gene, *GNMT*, encode both mRNAs and noncoding RNAs, and several *CNPY3*-*GNMT* RNAs are also protein-coding and noncoding (Figure 5), although we do not know whether the two-gene RNAs are derived from a readthrough, a *trans*-splicing, an unknown mechanism, or even a combination of different mechanisms. To many RNA experts, it may not be necessary to point out that a given cell or tissue type in a given situation may not express all the RNA variants, such as all the *CNPY3*, *GNMT* or *CNPY3*-*GNMT* variants. However, it is worth noting that, currently, there is no pellucid definition for noncoding RNA. Many researchers arbitrarily consider those RNAs whose largest ORF is smaller than 100 codons, i.e., 300 nucleotides, as noncoding [141,142,143,144,145], and further arbitrarily regard those RNAs with 200 or more nucleotides as long noncoding ones while those smaller than 200 nucleotides (which may encode more than 60 amino acids) as short ones, while some others only consider those RNAs encoding less than 30 amino acids as noncoding [42,43]. Obviously, this definition of “noncoding” ignores ample evidence proving that peptides much smaller than 99 amino acids may have biological functions [145,146,147,148,149,150,151,152,153,154,155], as has been described by us [38]. Since peptides as short as 11 amino acids still have important biological functions [147,148,149,150], even some short noncoding RNAs may have effects by producing small proteins. Therefore, it is comprehensible that some RNAs are classified as noncoding in the NCBI database but as protein-coding in the Ensembl database. For instance, the *STX16*-*NPEPL1* RNA (Gene ID: 100534593; 20q13.32) is predicated to be noncoding in the NCBI database (NR_037945.1) but to be coding in the Ensembl database (ENSG00000254995).

## 6. Two-Gene RNAs from Unknown Mechanism Make RNA Classification Difficult

Traditionally, RNAs are classified into the three categories of messenger RNA, transfer RNA, and ribosomal RNA. However, long mature RNAs can actually be categorized in different ways, such as using the RNA polymerase that synthesizes the RNA [38], but each classification method has its strengths and weaknesses. For example, based on whether or not an RNA has a corresponding parental gene in the nuclear or mitochondrial genome, RNAs can be dichotomized into two groups, i.e., (1) those that have a corresponding parental gene, i.e., have a genomic DNA parent; and (2) those that are produced at the RNA level without a genomic parent [6]. The former group includes not only all those RNAs that are clearly known to be derived from a readthrough mechanism as a subgroup, but also all RNAs that are transcribed from fusion genes formed due to genetic alterations [15,16,17,18], mostly discerned in genetic diseases and tumors [22,23,24], as another subgroup. It needs to be pointed out that for most two-neighboring-gene RNAs, examples being listed in Table 1, their derivation is unknown, in part because *trans*-splicing as a possible mechanism has not yet been ruled out and, therefore, cannot currently be sorted into the “readthrough” subgroup. The latter group lacks a genomic parent and is complex because it covers a variety of noncolinear RNAs, including those neighboring-gene RNAs from unknown mechanisms. Therefore, all methods of sorting that we can think of seem to become problematic once dealing with those RNAs containing sequences from two neighboring genes resulting from unknown mechanisms. Actually, it is even more problematic when dealing with mitochondrial RNAs that may form trimeras or even tetrameras, i.e., those RNAs containing sequences of three or four mitochondrial genes, as we once reported [65], because how these trimeras or tetrameras are yielded remains unknown. 

**Figure 6:**
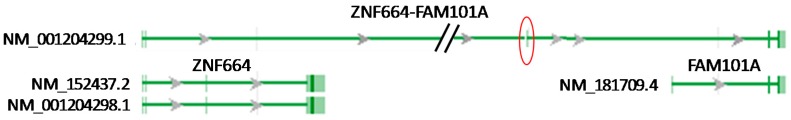
An image copied and modified from the NCBI showing that the *ZNF664*-*FAM101A* RNA contains one exon (in the red circle) derived from the very-long intergenic region, making this RNA more likely to be produced via a transcriptional-readthrough mechanism but not via a *trans*-splicing of a *ZNF664* transcript and a *FAM101A* transcript, although, theoretically, there may exist an unknown mechanism that can splice three transcripts (i.e., the *ZNF664*, the intergenic, and the *FAM101A* transcripts) into one mature RNA.

If a two-gene RNA is detected at high abundance in a situation wherein one of the two partner genes is undetectable, either the upstream or the downstream one, it may be a hint that a readthrough mechanism may underlie the production of the two-gene RNA, because the lack of one of the two partner transcripts makes *trans*-splicing impossible. Moreover, some two-gene RNAs contain exons from the intergenic region, such as the human *ZNF664*-*FAM101A* RNA produced from the 12q24.31 region (Figure 6). The existence of the intergenic-sequence-derived exon(s) makes it unlikely that the RNAs are produced via a *trans*-splicing of two individually transcribed RNA molecules, thus, indirectly supporting that the RNAs are derived from a readthrough mechanism. Nevertheless, uncontested experimental proof showing a readthrough event, including the existence of the not-yet-spliced precursor transcript and the relevant procedure, is still required for the claim that a two-gene RNA is engendered via readthrough. We should not assume that all two-neighboring-gene RNAs are produced by transcriptional readthrough simply because readthrough is common, while arbitrarily ruling out the possible involvement of *trans*-splicing that is also considered by other researchers to be a common event [15,66,67,68,69,70,71]. A caveat probably needs to be given that convincing experimental proof should require a non-RT and non-PCR approach to avoid technical spuriousness that may be created by these techniques [5,6,64,105,106,107,108,109,110,111,112,113,114,115,116,117,118,119,120,121,122,123,124,125,126,127,128,129,130,131,132,133,134,135], by using the cDNA protection assay established by us [105], the less sensitive RNA protection assay [136,137,138], or other approaches [110,111] as alternatives.

## 7. We Propose to Classify Long Mature RNAs into Four Types

In our opinion, long mature RNAs should be categorized based on the mechanism used to produce the RNA. There are two criteria for the mechanism, i.e., (1) whether or not the RNA has one single gene as the sole genomic parent and (2) whether or not the RNA is derived from *cis*-splicing of a single RNA transcript. By these criteria, all long mature RNAs that have been reported can be classified into four different types (Table 1). Those RNAs transcribed from already-annotated genes, which constitute the vast majority of long mature RNAs, are sorted into type I. It is essential to note that this type also includes those two-neighboring-gene RNAs that are clearly known to be derived from transcriptional readthrough or from fusion genes that are formed pathologically. This is because we regard each genomic locus encoding readthrough RNA as an unannotated, i.e., a newly-identified, gene, which in turn is because these unannotated genes do not show any difference from those already-annotated ones, pertaining to all transcriptional and posttranscriptional regulations. It goes without saying that these newly-identified genes should be annotated and assigned a name and a gene identification number (gene ID). Actually, the NCBI has already assigned a gene ID to each of those RNAs that contain sequences of two adjacent genes and named them simply by using a hyphen to link the names of the two genes, with examples shown in Table 1. We suggest to the RNA research fraternity to follow the NCBI’s nomenclature to annotate all those, and only those, RNAs that are clearly known to be derived from a readthrough mechanism. However, most of those two-neighboring-gene RNAs that have been reported in the literature or listed in the NCBI database have not yet been confirmed to be derived via this mechanism and, thus, should not be grouped into this category at the moment, in our opinion. Therefore, to accommodate those RNAs for which derivation is not yet known, we temporarily put them into type II. Here, “temporarily” means that they should eventually be recategorized into either type I if a readthrough is later confirmed, or into a new type if a *trans*-splicing event is confirmed or a new mechanism is identified. Those noncolinear RNAs that are not two-gene ones, such as the aforementioned *KLK4* RNA variant containing both sense and antisense [75] as well as the *ERα* RNA variants that contain duplicated exons [54,55,56], are all grouped into type III. It remains possible that a *trans*-splicing or a currently-unknown mechanism may account for the formations of this type of RNA. Those RNAs that contain sequences of two genes on different chromosomes and for which *trans*-splicing has been claimed as a source, such as the *JAZF1*-*JJAZ1* chimeric RNA that was reported to be derived via a mechanism mimicking *trans*-splicing [78,81], are authentic chimeric RNAs and are classified into type IV. 

## 8. Do *Trans*-Splicing and Authentic Chimeric RNAs Really Exist in Human Cells?

Although we are aware of a handful of RNAs in human cells that have been reported to be chimeric RNAs formed via *trans*-splicing [25,54,55,56,75,76,78,87,88,89,90,91,137,156], and have grouped them into type IV in Table 2, we still doubt (1) whether *trans*-splicing really exists and, thus, (2) whether *trans*-splicing-derived authentic chimeric RNAs truly exist, in human cells. We have several lines of thought that lead us to these suspicions:

1. The number of *cis*-splicing events and *cis*-splicing derived RNAs in human cells are numerous, and *trans*-splicing is very common in evolutionarily-low organisms [157,158,159,160], whereas reported *trans*-splicing events in human cells have so far been very few. Therefore, it seems to us that *trans*-splicing may have undergone regression during evolution towards higher organisms, although we still need to determine whether *trans*-splicing has become defunct in healthy humans and whether it reappears during carcinogenesis, which would be considered an atavism, i.e., a reverse-evolutionary process.
2. Most, if not all, published studies that claim the observation of *trans*-splicing in human cells do not provide us with procedural and mechanistic details of the splicing. Therefore, we still know very little about it, although *cis*-splicing is well-characterized in human cells and *trans*-splicing is well characterized in evolutionarily-lower organisms. For example, although we do know that a large number of proteins are involved in *cis*-splicing, we do not know how many proteins are involved in *trans*-splicing and what these proteins are in human cells. After more than a decade since the initial publications on many chimeric RNAs and other noncolinear RNAs that are believed to be derived from *trans*-splicing, few follow-up studies, either by the initial reporters or by other researchers, have been published on the procedural and mechanistic details of the *trans*-splicing per se and of how the splicing leads to the formation of chimeras or other noncolinear RNAs in human cells.
3. If *trans*-splicing does exist in human cells as a mechanism for chimeric RNA formation, we should see more of those chimeras with sequences of two genes that are on the same chromosome but are farther away from each other, too far away for transcriptional readthrough to occur. However, the fact is that two-distant-gene chimeras, if they exist, are rare, which provides indirect evidence against the true existence of a *trans*-splicing mechanism.
4. Yu et al. once tried to validate many reported noncolinear RNAs and suggested that 50% of them are artifacts produced in vitro [161]. This high rate of spuriousness identified by a single study suggests to us that more stringent vindication is required for authentication of the remaining 50%.

## 9. Concluding Remarks

Tens of thousands of so-called chimeric RNAs in human cells have been reported in the literature or deposited in different databases, but many of them may be technical artifacts produced during RT or PCR that is part of the high-throughput RNA sequencing technology [5,6,64,105,106,107,108,109,110,111,112,113,114,115,116,117,118,119,120,121,122,123,124,125,126,127,128,129,130,131,132,133,134,135]. Most of these chimeras contain sequences of two adjacent genes on the same chromosome and are generally considered to be derived via transcriptional readthrough, but for many of them this remains a reasonable assumption awaiting uncontentious evidence, in part because *trans*-splicing is still a possible mechanism. We agree on the readthrough assumption but regard those genomic loci that are transcriptionally read through as previously unidentified, or newly identified, genes waiting for annotation and characterization. To reiterate, we do not consider readthrough-derived RNAs as chimeras, because readthrough genomic loci reflect the phenomenon of “a gene contains gene(s)” or “gene(s) within a gene” seen in the human genome, and show no difference from the 20,000 human genes and from all fusion genes formed due to genetic alterations. Recapitulated more categorically, there is no difference among unannotated, already-annotated, and fusion genes appertaining to their transcriptional, posttranscriptional, translational, and posttranslational regulations. Therefore, we find no reason to call readthrough RNAs chimeras. We define authentic chimeric RNAs as those formed at the RNA level without one corresponding gene as the sole genomic parent. *Trans*-splicing is the only possible mechanism known so far to be accountable for the formation of such authentic chimeras and other forms of noncolinear RNAs, and probably for the formation of some two-neighboring-gene RNAs as well. However, we doubt the true existence of *trans*-splicing and, thus, the true existence of authentic chimeric RNAs, in human cells, in part because very few RNAs that might be derived from *trans*-splicing have been reported so far, and, for these RNAs, there is a lack of procedural and mechanistic details of the presumed *trans*-splicing. Although we sort long mature RNAs into four different types to accommodate all reported ones, there probably is only one single type, i.e., type I in Table 2, because those in our type II will eventually be regrouped into type I while those in our types III and IV may not really exist, by our speculation. In our opinion, partly because readthrough-derived RNAs are commonly considered as chimeras in the RNA research province, characterization of their parental genes has largely been forgotten, which in turn impedes our understanding of these newly-identified genes. Therefore, it is imperative to stop considering these RNAs as chimeras and, instead, to characterize, as we have for many other genes, their parental genes at all transcriptional, posttranscriptional, translational, and posttranslational levels, with emphasis on their alternative *cis*-splicing. Moreover, it is imperative to determine whether *trans*-splicing really occurs in human cells. If it does not exist, then those two-neighboring-gene RNAs cannot be derived from it and, thus, are more likely to come from a transcriptional readthrough. On the other hand, if it really exists, those RNAs thought to be derived from *trans*-splicing are likely authentic chimeras and many more authentic ones may be awaiting our discovery.

## Figures and Tables

**Table 1:** Some two-neighboring-gene RNAs of the human origin documented in the NCBI database.

Name	Gene ID *	Location	Coding or not	Name	Gene ID	Location	Coding or not
*MROH7-TTC4*	100527960	1p32.3	noncoding	*DNAAF4-CCPG1*	100533483	15q21.3	noncoding
*GJA9-MYCBP*	100527950	1p34.3	noncoding	*ST20-MTHFS*	100528021	15q25.1	coding
*CENPS-CORT*	100526739	1p36.22	both **	*C15orf38-AP3S2*	100526783	15q26.1	coding
*PMF1-BGLAP*	100527963	1q22	coding	*SLX1A-SULT1A3*	100526830	16p11.2	noncoding
*TSNAX-DISC1*	100303453	1q42.2	noncoding	*SLX1B-SULT1A4*	100526831	16p11.2	noncoding
*HSPE1-MOB4*	100529241	2q33.1	coding	*BOLA2-SMG1P6*	107282092	16p11.2	both
*ABHD14A-ACY1*	100526760	3p21.2	coding	*PKD1P6-NPIPP1*	105369154	16p13.11	noncoding
*ARPC4-TTLL3*	100526693	3p25.3	coding	*CORO7-PAM16*	100529144	16p13.3	coding
*FAM47E-STBD1*	100631383	4q21.1	coding	*CKLF-CMTM1*	100529251	16q21	coding
*TMED7-TICAM2*	100302736	5q22.3	coding	*TVP23C-CDRT4*	100533496	17p12	both
*CNPY3-GNMT*	107080644	6p21.1	both	*RNASEK-C17orf49*	100529209	17p13.1	noncoding
*RPS10-NUDT3*	100529239	6p21.31	coding	*TNFSF12-TNFSF13*	407977	17p13.1	coding
*PPT2-EGFL8*	100532746	6p21.32	noncoding	*SENP3-EIF4A1*	100533955	17p13.1	noncoding
*ATP6V1G2-DDX39B*	100532737	6p21.33	noncoding	*RAD51L3-RFFL*	100529207	17q12	noncoding
*MSH5-SAPCD1*	100532732	6p21.33	noncoding	*PTGES3L-AARSD1*	100885850	17q21.31	coding
*BLOC1S5-TXNDC5*	100526836	6p24.3	noncoding	*NME1-NME2*	654364	17q21.33	both
*EEF1E1-BLOC1S5*	100526837	6p24.3	noncoding	*TBC1D3P1-DHX40P1*	653645	17q23.1	noncoding
*URGCP-MRPS24*	100534592	7p13	coding	*TEN1-CDK3*	100529145	17q25.1	noncoding
*ATP5J2-PTCD1*	100526740	7q22.1	coding	*RPL17-C18orf32*	100526842	18q21.1	coding
*C7orf55-LUC7L2*	100996928	7q34	coding	*PPAN-P2RY11*	692312	19p13.2	coding
*C10orf32-AS3MT*	100528007	10q24.32	noncoding	*RAB4B-EGLN2*	100529264	19q13.2	noncoding
*TMX2-CTNND1*	100528016	11q12.1	noncoding	*MIA-RAB4B*	100529262	19q13.2	noncoding
*KCNK4-TEX40*	106780802	11q13.1	noncoding	*FKBP1A-SDCBP2*	100528031	20p13	noncoding
*RBM14-RBM4*	100526737	11q13.2	coding	*SYS1-DBNDD2*	767557	20q13.12	noncoding
*HSPB2-C11orf52*	100528019	11q23.1	noncoding	*SLMO2-ATP5E*	100533975	20q13.32	noncoding
*BLOC1S1-RDH5*	100528022	12q13.2	noncoding	*STX16-NPEPL1*	100534593	20q13.32	noncoding
*ZNF664-RFLNA*	100533183	12q24.31	coding	*SPECC1L-ADORA2A*	101730217	22q11.23	noncoding
*BCL2L2-PABPN1*	100529063	14q11.2	coding	*PIR-FIGF*	100532742	Xp22.2	noncoding
*CHURC1-FNTB*	100529261	14q23.3	coding	*RPL36A-HNRNPH2*	100529097	Xq22.1	coding
*SERF2-C15orf63*	100529067	15q15.3	noncoding				

*: “Gene ID” means gene identification number. **: “Both” means that some RNA variant(s) are protein-coding while some other(s) are noncoding.

**Table 2:** Classification of long mature RNAs.

#	Transcript Mechanism	Genetic Base	RNAs
I	Well characterized	With an annotated or unannotated (including reathrough) gene as a base	Classical mRNAs and noncoding RNAs
			Circular RNAs
		With a fusion gene as a DNA base	Fusion RNAs
II	Unknown	With or without a DNA base?	RNAs with neighboring-genes’ sequences
III	Less known	Without a DNA base	RNAs with sense and antisense sequences
			RNAs with duplicated exons
IV	Unknown	With two genes as bases	Authentic chimeric RNAs

Note: “Transcript mechanism” indicates the regulatory mechanisms for the transcription and posttranscription, including *cis*-splicing. Readthrough RNAs are considered to be derived from unannotated genes and thus grouped into type I.
